# Trans-apical catheter ablation of ventricular tachycardia in a patient with metallic aortic and mitral valves

**DOI:** 10.1186/s13019-024-02478-4

**Published:** 2024-01-20

**Authors:** Ali Bozorgi, Saeed Sadeghian, Entezar Mehrabi Nasab

**Affiliations:** 1 Department of Cardiology, School of Medicine, Tehran Heart Center, Tehran University of Medical Sciences, North Kargar Street, Tehran, Iran; 2 Department of Cardiology, School of Medicine, Valiasr Hospital, Zanjan University of Medical Sciences, Zanjan, Iran

**Keywords:** Ventricular tachycardia, Radiofrequency catheter ablation, Heart valve diseases

## Abstract

We present a patient with a history of heart failure and metallic aortic and mitral valves surgeries, who required ablation for a drug-refractory left ventricular tachycardia. But the metallic valves prohibited the insertion of catheters via retrograde or via trans-septal approaches. Therefore, we decided to perform catheter ablation by direct left ventricle puncture through a minithoracotomy. The arrhythmia was successfully ablated via of trans-apical approach and did not recur at six months follow-up.

**Supplementary Information:**

The online version contains supplementary material available at 10.1186/s13019-024-02478-4.

## Key points

- Introduce an alternative route for VT ablation in patients with mechanical aortic and mitral valves.
- Emphasis on the role of heart team in the management of complex life-threatening arrhythmia.

**Supplementary Information:**

The online version contains supplementary material available at 10.1186/s13019-024-02478-4.

## Introduction

In patients with a history of device implantation, incessant ventricular tachycardia (VT) leads to frequent discharge of the Implantable cardioverter defibrillator (ICD), causing pain and anxiety for the patient. When antiarrhythmic drugs are ineffective, catheter ablation is the only treatment option. The vast majority of VAs originate from the endocardium [1]. The main challenge for radiofrequency ablation of VT is access to the endocardium. Typically, access to the left ventricular endocardium is possible with a transseptal atrial approach or a transaortic approach. Catheter ablation is very challenging in patients with mechanical prosthetic mitral and aortic valves because conventional access to the left ventricle (LV), either via a transseptal atrial or retrograde aortic approach, is not possible. Because the use of the transseptal method can cause damage to the mitral valve, and the use of the retrograde aortic approach can cause damage to the aortic valve [2–5]. Of course, in patients with mechanical prosthetic mitral and aortic valves, another method is to create a puncture in the wall between the right atrium (RA) and the left ventricle (LV) with the transseptal method [6]. But this method is very difficult and requires a high level of expertise and sufficient facilities.

The appropriate solution in a patient with double mechanical heart valves is direct left ventricle puncture through a minithoracotomy [7] In this article, we have reported a case of trans-apical approach in a patient with double mechanical heart valves.

## Case report

**Fig. 1:**
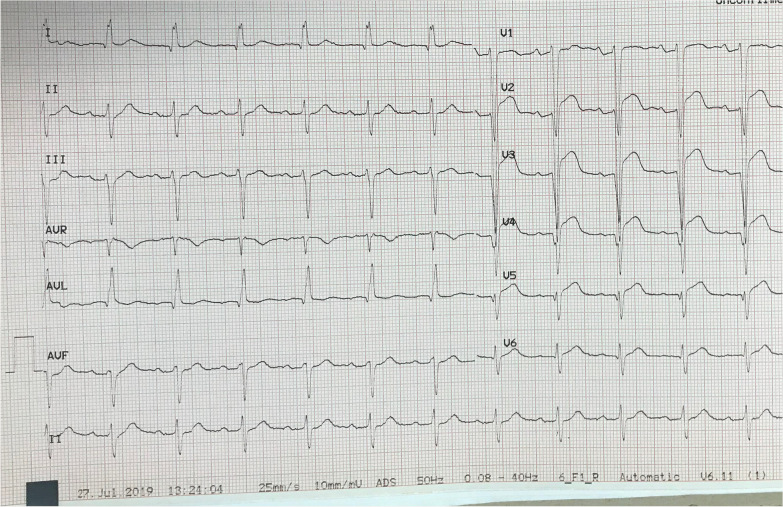
Baseline electrocardiography at the first-time admission. The rhythm is normal sinus rhythm along with leftward axis deviation, fragmented QRS morphology in lead I, and pathologic Q wave in V_1_-V_6_, along with inverted T wave in I and aVL

**Fig. 2:**
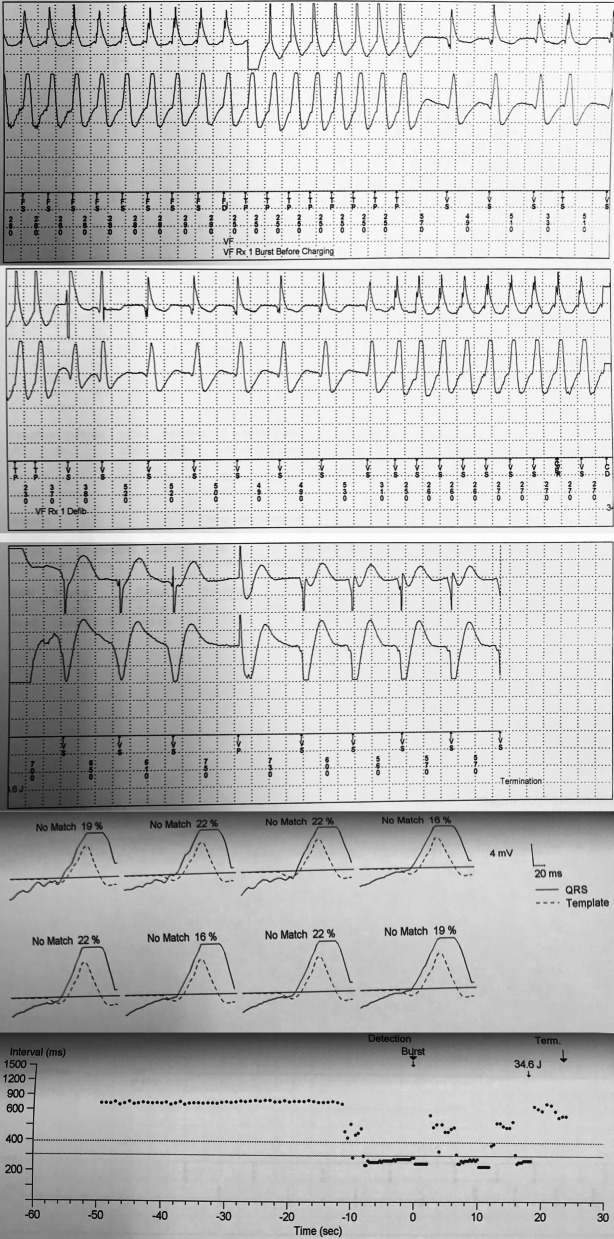
ICD interrogation for multiple episodes of ventricular tachyarrhythmia

**Fig. 3:**
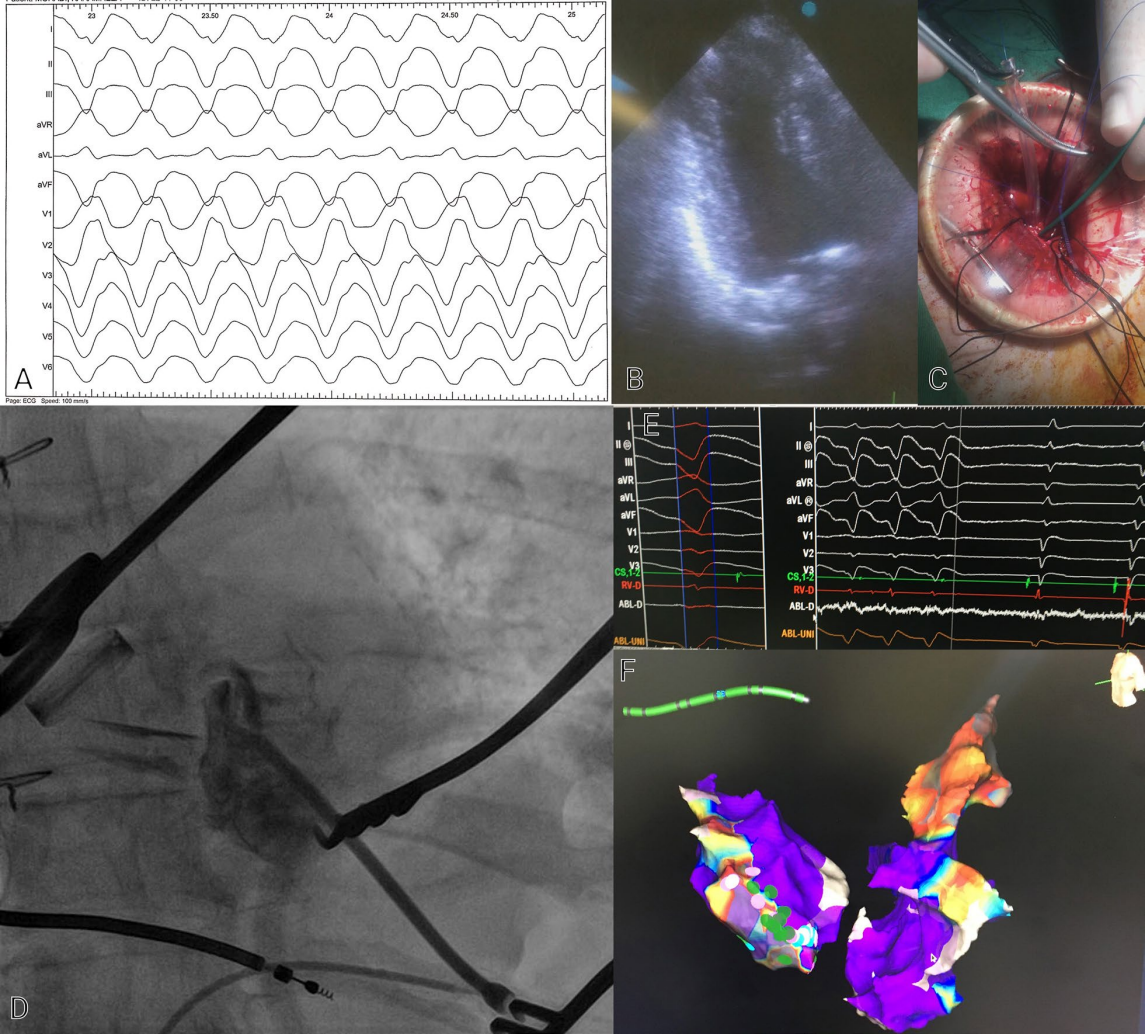
EPS, transapical access and mapping of ventricular tachycardia, and RF ablation. EPS was done and showed easily inducible VT, originated mainly from posterior aspect of LV apex **A**. Echocardiography showed aneurysmal LV apex **B** and the surgeon made an incision in the left thorax with the guide of echocardiography at LV apex **C** and inserted the ablation catheter using the modified Seldinger approach. Left ventriculography was done to prevent inadvertent contact between the ablation catheter and the metallic prosthetic valves **D**. RF ablation was done and VT was terminated **E**. Voltage mapping of LV aneurysm and epicardium along with RF ablation spots

A 70-year-old man was referred to our center due to recurrent episodes of VT causing multiple ICD shocks. He had a history of metal mechanical aortic and mitral valve replacement 24 years prior to his presentation. The patient had a history of previous MI and he had three vessels coronary disease (3VD) and was not CABG or PCI candidate and was under medical therapy. He had a secondary prevention ICD implanted 4 years ago. Unfortunately, there was no information on patient’s presentation at the time of ICD implant. Despite optimization of his medical therapy, including the addition of Amiodarone and Mexiletine for 6 months, he continued to have repetitive episodes of appropriate ICD shocks. The patient was admitted to our center for catheter ablation. Complete laboratory tests were performed. His Electrocardiogram (ECG) is shown in Fig. 1. Transthoracic echocardiogram showed an aneurysmal LV apex and severely reduced LV systolic function with an LV-ejection fraction (EF) of 20%. Prosthetic valves’ gradients were acceptable, and no paravalvular leak was noted. ICD interrogation showed frequent episodes of VT with majority of them successfully treated with Anti-tachycardia pacing (ATP); however, some requiring shocks (Fig. 2). Twelve lead ECG of the VT was not available. An electrophysiological study (EPS) was performed. Monomorphic VT a Right bundle branch block (RBBB) pattern with transition in V2-V3 (or negative concordance), superior axis, negative in I consistent with an exit site in the postero-lateral LV apex was easily inducible (Fig. 3A). Given the presence of mechanical valves in mitral and aortic positions, retrograde aortic and transseptal LV access were not feasible. Between RA-LV could be a good access but due to lack of evidence and very difficult septostomy from there, this method was not preferred. After a multidisciplinary discussion, it was decided to attempt VT ablation via a trans-apical approach.The procedure was performed under general anesthesia. A quadripolar right ventricular (RV) catheter was placed in the RV apex via the right femoral vein. Through a left anterior incision, and with guidance of echocardiogram, LV apex was exposed by surgeon, and an 8-Fr sheath was advanced into the LV (Fig. 3B, C and D). Initial anatomical and voltage mapping, using Abbott’s Ensite Precision system, demonstrated a large apical scar and aneurysm. It was consistent with the aneurysmal portion of the LV apex demonstrated on echocardiography. VT with similar morphology to the patient’s clinical VT, was induced spontaneously (Fig. 3E). During the procedure, the sheath and catheter were accidently pulled out of LV cavity; interestingly there was no significant bleeding and no apparent blood jet.

Catheter was re-inserted through the same puncture site and to ensure better maneuverability a sheath was not used. After defining the scar area with geometry and substrate mapping, Pace mapping was done to resemble morphology of clinical VT. VT was spontaneously induced during mapping and mapping was continued during VT. Patient was hemodynamically stable during VT and tolerated the mapping well; however, due to procedure’s critical setting, it was decided to defer activation and entrainment mapping and minimize the procedure time.

Due to ischemic CMP and low chance of success by epicardial approach and probable adhesions for previous sternotomy, we decided to go straight to endocardium. After endocardial mapping, epicardial mapping was also performed but during epicardial mapping approach not found good signal. Finally, suitable signals for ablation were found with the endocardial mapping approach and then With use of FlexAbility irrigated ablation catheter (Abbot medical), several radiofrequency (RF) lesions were applied on the border zone of scar (ablation setting power: 30–35-W, temperature: 42ºC, impedance drop to ≤ 15Ω) and the scar area was successfully electrically isolated. Up to thirty minutes after the ablation procedure, VT was not inducible with either burst pacing or programmed stimulation andS3 protocol (500/ 350/ 250).While suturing the LV apex, one episode of slow VT occurred which was terminated with burst pacing. The VT morphology was not similar to clinical VT. Access site was closed, and a Jackson-Pratt drain was inserted. The early post-procedure course was satisfactory, without any significant complications. The patient was on Warfarin which was bridged with Heparin. Bleeding was less than 250 cc, but due to anticoagulation needed for prosthetic valves, we decided to put the drain longer.

ICD interrogation confirmed no further VT episode during patient’s admission. He was discharged home on amiodarone 200 mg (mg) Twice a day (BID), mexiletine 200mg BID, captopril 6.25mg TDS, metoprolol succinate 23.75mg once daily (OD), atorvastatin 20mg OD, Acetylsalicylic acid (ASA) 80mg OD. Also, due to pericardial manipulation in the time of surgery, a course of anti-inflammatory drugs was initiated [Ibuprofen 400mg three times a day (TID), and Colchicine 0.5mg OD]. Patient symptoms relieved in 3 days. No further VT episodes were detected on ICD interrogation at 1, 3 and 6 months of follow up. After 6 months, we discontinued mexiletine and continued amiodarone 200 mg/day for the patient.

## Discussion

There are a few reports of trans-apical approach for accessing the left-heart structures, including left atrium (LA) and LV, for treatment of both atrial and ventricular tachyarrhythmia in animal models and human patients [8, 9]. The trans-apical approach was also used for VT ablation in toddlers after viral myocarditis [10] or cardiogenic shock [11]. In patients with only mechanical aortic valve, trans-septal approach is an option for catheter ablation of ventricular arrhythmias; however, this approach and retrograde aortic LV access are not feasible in the presence of concurrent mechanical aortic and mitral valves, due to the risk of catheter entrapment between the prosthetic valve leaflets resulting in catastrophic complications. We present a case of successful trans-apical VT ablation with minimal complications. Due to high risk of thrombotic events, the procedure was performed with full anticoagulation which resulted in prolonged mild bleeding and retaining of the drain.

The trans-apical approach is usually used as the last resort for tachyarrhythmia ablation. For patients with mitral valve (MV) and aortic valve (AV) mechanical valve and refractory VT; however, treatment options are limited. These patients may benefit from trans-apical ablation. Recent case reports suggest that this approach may be the preferred route in fully anti-coagulated patients [e.g., subjects on extracorporeal membrane oxygenation (ECMO)] because of direct visualization of the incised region [10]. Not only does this approach provide access and better catheter maneuverability for VT ablation in these patients, but it also offers access for epicardial ablation.

The trans-apical approach has limitations. Primarily, it demands individual expertise and meticulous coordination among all members of the heart-team. Previous cardiac surgeries may cause pericardial adhesion and fibrosis. The trans-apical approach may also damage the apical structures (RV apex, left anterior descending artery) and cause life-threatening complications. External and internal bleeding from the LV apex is the most reported complication and the surgeon should be careful and try to prevent and control bleeding, mainly through good hemostatic techniques, not by reversing anti-coagulation.

We had some limitations in this study. We did not have ICE at that time and we could not do this procedure for our patient and therefore, we had no enough data about this part. Also, we did not have experience doing RA- > LV septostomy and we had to do this according to reported processes.

## Conclusion

With increase in the prevalence of heart failure and valvular surgeries, the trans-apical approach for VT ablation will likely be used more frequently in the forthcoming years. With proper coordination among the heart-team members along with meticulous case selection this approach may carry a high rate of success and a low rate of complications, even in patients with multiple comorbidities.

### Supplementary Information

**Additional file 1**: Video of the process of catheter ablation of VT in a patient with metallic aortic and mitral valves.

## Data Availability

Not applicable.

## References

[1] Eckart RE, Hruczkowski TW, Tedrow UB, Koplan BA, Epstein LM, Stevenson WG (2007). Sustained ventricular tachycardia associated with corrective valve surgery. Circulation.

[2] Gao M-Y, Zeng L-J, Li X-X, Tian Y, Pi-Xiong Su, Yang X-C, Liu X-P (2020). Ablation of ventricular tachycardia by direct left ventricle puncture through a minithoracotomy after double valve replacement: a case report and literature review. J Int Med Res.

[3] Hawson J, Kalman J, Goldblatt J, Anderson RD, Watts T, Hardcastle N, Siva S, Kumar S, Lee G (2022). From minimally to maximally invasive; VT ablation in the setting of mechanical aortic and mitral valves. J Cardiovasc Electrophysiol.

[4] Bennett RG, Garikapati K, Anderson RD, Silva K, Campbell T, Kotake Y, Turnbull S, Tonchev I, Lee G, Kalman J, Kumar S (2022). Clinical, electroanatomic and electrophysiologic characterization and outcomes of catheter ablation for ventricular tachycardia following valvular intervention. J Cardiovasc Electrophysiol.

[5] Aras D, Topaloglu S, Ozeke O, Ozcan F, Cay S, Golbasi Z (2019). Percutaneous interventricular septal access guided by subcostal echocardiography and fluoroscopy for ventricular tachycardia ablation in a patient with aortic and mitral mechanical valves. J Innov Card Rhythm Manag.

[6] Pasquale S, Matthew C (2021). Outcomes of percutaneous trans-right atrial access to the left ventricle for catheter ablation of ventricular tachycardia in patient with mechanical aortic and mitral valves. JAMA Cardiol.

[7] Imnadze G, Hofmann S, Warnecke H, Zerm T (2016). Epicardial ablation of ventricular tachycardia using pericardioscopy through submammary minimal thoracotomy. Interact Cardiovasc Thorac Surg.

[8] Reents T, Stilz S, Herold U, Deisenhofer I (2014). Transapical access for catheter ablation of left ventricular tachycardia in a patient with mechanical aortic and mitral valve prosthesis. Clin Res Cardiol.

[9] Hsieh CH, Thomas SP, Ross DL (2010). Direct transthoracic access to the left ventricle for catheter ablation of ventricular tachycardia. Circ Arrhythm Electrophysiol.

[10] Benhayon D, Cogan J, Scholl F, Latson L, Alkon J, Young M-L (2016). Left ventricular tachycardia ablation in a toddler via a transapical approach: a new tool for the armamentarium. HeartRhythm Case Report.

[11] Koutbi L, Chenu C, Mace L, Franceschi F (2015). Ablation of idiopathic ventricular tachycardia arising from posterior mitral annulus in an 11-month-old infant by transapical left ventricular access via median sternotomy. Heart Rhythm.

